# Cardiac injury progression in children with multisystem inflammatory syndrome associated with SARS-CoV-2 infection: a review

**DOI:** 10.3389/fped.2024.1348016

**Published:** 2024-03-06

**Authors:** Song Su, Wandong Hu, Xiao Chen, Ying Ren, Yi Lu, Jianguo Shi, Tong Zhang, Huan Zhang, Meng Wang, Yaping Wang, Fen Zhao, Ruifeng Jin, Yong Liu, Hongwei Zhang, Guohua Liu

**Affiliations:** ^1^ Epilepsy Center, Children’s Hospital Affiliated to Shandong University, Jinan, Shandong, China; ^2^ Epilepsy Center, Jinan Children's Hospital, Jinan, Shandong, China; ^3^ Department of Ophthalmology, Children's Hospital Affiliated to Shandong University, Jinan, Shandong, China; ^4^ Department of Ophthalmology, Jinan Children's Hospital, Jinan, Shandong, China

**Keywords:** severe acute respiratory syndrome coronavirus 2, children, multisystem inflammatory syndrome, cardiac injury, coronavirus disease 2019

## Abstract

The symptoms and signs of infection caused by severe acute respiratory syndrome coronavirus 2 (SARS-CoV-2) are milder in children than in adults. However, in April 2020, British pediatricians first reported that coronavirus disease 2019 (COVID-19) may present as multisystem inflammatory syndrome in children and adolescents (MIS-C), similar to that observed in Kawasaki disease. MIS-C can be associated with multiple systemic injuries and even death in children. In addition to digestive system involvement, cardiac injury is prominent. This article reviews the pathogenesis, clinical manifestations, and treatment of cardiac injury caused by MIS-C, which may help clinicians in early diagnosis and timely commencement of treatment.

## Introduction

Coronavirus disease 2019 (COVID-19) caused by severe acute respiratory syndrome coronavirus 2 (SARS-CoV-2) has led to high morbidity and mortality worldwide, which has generated significant global concern. The World Health Organization (WHO) declared COVID-19 a pandemic on March 11, 2020. COVID-19 is less severe in children than in adults because of the lower expression of angiotensin-converting enzyme 2 (ACE2) receptors, a poorly developed immune system, and the slightest possibility of inflammatory cytokine storms causing limited virus invasion, thereby avoiding large-scale outbreaks in children. However, severe cases of COVID-19 in children have been reported in Europe and the United States since April 2020, with symptoms and signs similar to those of incomplete Kawasaki disease or toxic shock syndrome (1–5). The Centers for Disease Control and Prevention (CDC) and the WHO named it a multisystem inflammatory syndrome in children and adolescents (MIS-C), with persistent high fever, digestive tract symptoms, shock, cardiac dysfunction, nervous system disorders, and other multi-organ injuries. Most children develop digestive system disorders, followed by cardiac injury; nonetheless, the manifestations of cardiac injury are variable and potentially fatal. This review focuses on the pathogenesis, clinical manifestations, and treatment of MIS-C and cardiac injury in children.

## SARS-CoV-2 structure

**Figure 1:**
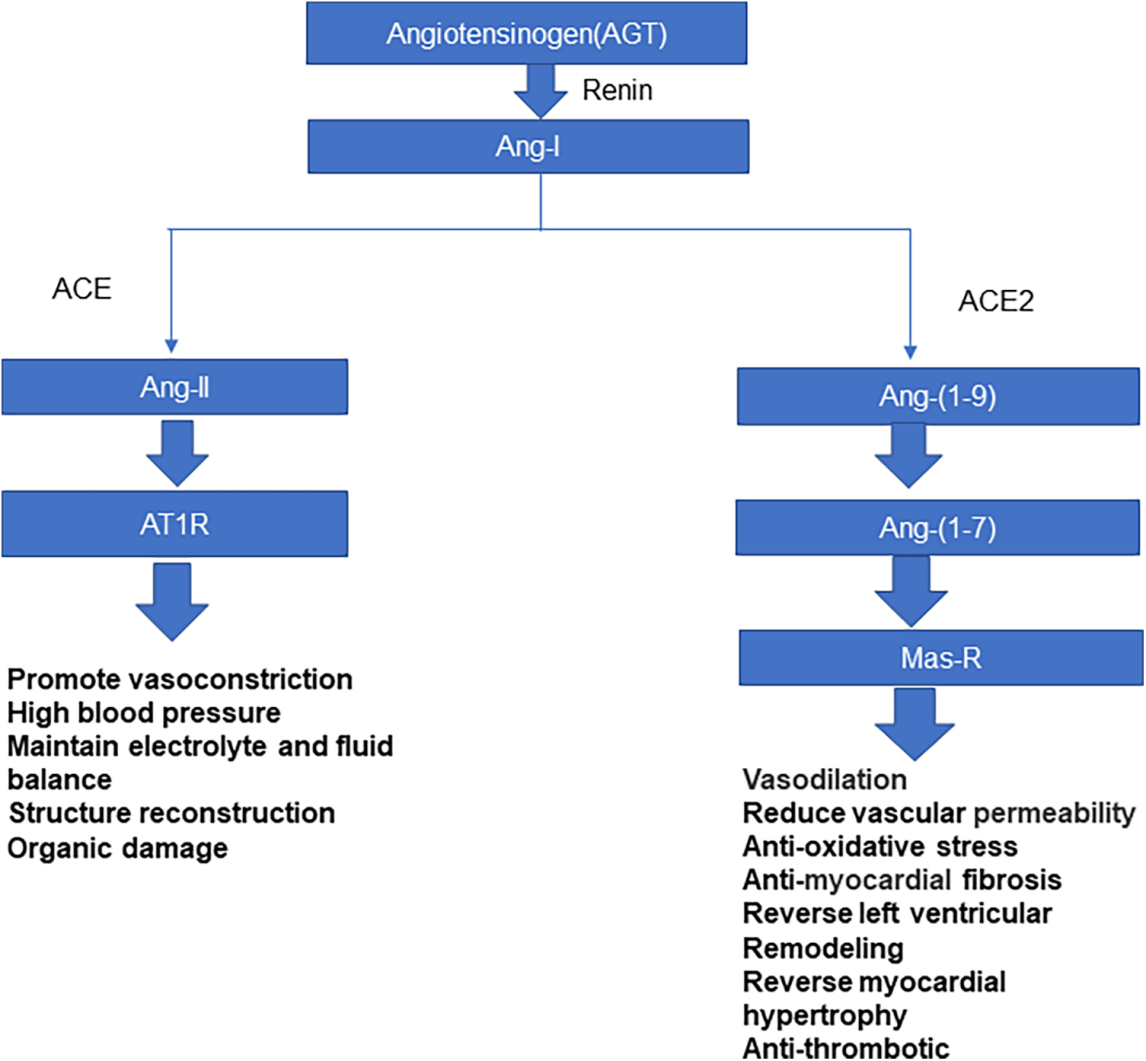
Mechanism of the angiotensin-aldosterone-renin system.

Coronavirus is a single-stranded RNA virus, which is divided into four genera: α, β, γ, and δ (6). Coronavirus β includes SARS-CoV, SARS-CoV-2, and the Middle East respiratory syndrome coronavirus. SARS-CoV-2 is transmitted mainly through inhalation of respiratory droplets from infected people or through contact with virus-contaminated surfaces. The spike glycoprotein, S protein, present on the surface of the coronavirus makes this series of viruses appear typical of coronaviruses microscopically (6). Coronavirus entry into host cells is regulated by S protein, which is divided into S1 and S2 subunits. The S1 subunit includes amino-terminal domain and carboxy-terminal domain (CTD); The CTD recognizes and binds to receptors, also known as receptor-binding domain (RBD). The entry of coronavirus into host cells is a complex process that requires the synergistic effect of binding with receptors and catalytic treatment of S protein to promote virus and host cell fusion (7, 8). ACE2 is a necessary enzyme in the renin-angiotensin system (RAS), which can effectively maintain RAS balance (9). ACE2 is a type-I membrane protein. Full-length ACE2 consists of a peptidase domain (PD) and a collagen-like domain. ACE2 mainly regulates angiotensin, controls vasoconstriction and blood pressure, and protects the cardiovascular system and many other tissues and organs (8, 10, 11) (Figure 1). Hamming et al. (12) verified that ACE2 is highly expressed in many other tissues and cells, such as myocardium, vascular endothelial cells, proximal renal tubules, bladder epithelial cells, and human lung and intestinal epithelial cells. Li et al. showed that ACE2 is the receptor of severe acute respiratory syndrome coronavirus (SARS-CoV) in 2003, and Yan et al. found that it is the receptor of SARS-CoV-2 in 2020 (7, 13, 14). The S protein on SARS-CoV-2's surface has a high affinity for ACE2. Novel coronavirus enters the host cell mainly through S protein on the virus surface binding to the ACE2 receptor (15); the spike RBD of SARS-CoV-2 binds to the ACE2 PD to form an RBD-PD complex, allowing the virus to enter the host cell (7). The heart is a high-risk target for SARS-CoV-2 infection. Most children with MIS-C have cardiac injury; malignant arrhythmias and heart failure can occur in severe cases, which is closely related to case mortality (16).

## Epidemiological characteristics and clinical manifestations of MIS-C

Multiple research teams have conducted meta-analyses of MIS-C caused by SARS-CoV-2 infection (1, 3, 4, 17), finding that MIS-C is found in children of all ages, mostly 6–15 years old, with a male to female ratio approximating 1:1; black children are at greater risk than Asians; most MIS-C appears 4–6 weeks after SARS-CoV-2 infection in children (18); and respiratory, circulatory, renal, digestive, and nervous system injuries can occur in MIS-C. Among these, digestive system involvement is prominent, followed by cardiovascular system involvement. Statistics showed that 50%–90% of patients had digestive tract disorders, 50%–81% had cardiogenic shock, 53%–80% had cardiovascular diseases, 21%–70% had respiratory disorders, 22%–52% had acute renal injuries, and nervous system disorders occurred in 12%–57%. Most patients with MIS-C have a high incidence of gastrointestinal tract symptoms, such as vomiting and diarrhea (17, 19). The WHO, CDC, and the Royal College of Pediatrics and Child Health have established corresponding standards for the definition and diagnosis of inflammatory response syndrome in children, as shown in Table 1 (20).

**Table 1:** Standards and definitions of the inflammatory response syndrome in children.

Criteria	World Health Organization	Centers for Disease Control and Prevention (United States)
Age	0–19 years	<21 years
Fever	Fever for ≥3 day	Temperature ≥38.0°C for ≥24 h or subjective fever for ≥24 h
Clinical symptoms	At least 2 of the following: 1. Rash or bilateral non-purulent conjunctivitis or mucocutaneous inflammation signs (oral, hands, or feet) 2. Hypotension or shock 3. Features of myocardial dysfunction, pericarditis, valvulitis, or coronary abnormalities (including ECHO findings or elevated troponin/ NT-proBNP) 4. Evidence of coagulopathy (by prothrombin time, activated partial thromboplastin time, elevated D-dimers) 5. Acute gastrointestinal problems (diarrhea, vomiting, or abdominal pain)	Both of the following: 1. An individual aged <21 years presenting with fever, laboratory evidence of inflammation, and evidence of clinically severe illness requiring hospitalization 2. Multisystem (>2) organ involvement (cardiac, kidney, respiratory, hematologic, gastrointestinal, dermatologic, or neurological)
Inflammation	Elevated inflammation markers, including any of the following: 1. ESR 2. CRP 3. Procalcitonin	Laboratory evidence of inflammation including, but not limited to, one or more of the following: 1. CRP↑ 2. ESR↑ 3. Fibrinogen↑ 4. Procalcitonin↑ 5. D-dimer↑ 6. Ferritin↑ 7. LDH↑ 8. IL-6↑ 9. Neutrophilia 10. Lymphopenia 11. Hypoalbuminemia
Link to SARS-CoV-2	Evidence of COVID-19 by the following: 1. Positive PCR; 2. Positive antigen test; 3. Positive serology; or 4. Likely COVID-19 contact	Current or recent findings of the following: 1. Positive PCR; 2. Positive serology; 3. Positive antigen test; or 4. COVID-19 exposure within the previous 4 weeks
Exclusion	No other obvious microbial cause of inflammation, including bacterial sepsis, or staphylococcal or streptococcal shock syndromes.	No alternative plausible diagnoses

NT-proBNP, N-terminal pro brain natriuretic peptide; ESR, erythrocyte sedimentation rate; CRP, C-reactive protein; LDH, lactate dehydrogenase; IL-6, interleukin-6.

## Pathophysiological mechanism

### Immune-mediated injury

The clinical characteristics of an inflammatory response syndrome, laboratory test findings, and the temporal association with SARS-CoV-2 infection support the hypothesis that SARS-CoV-2 infection causes immune-mediated damage to tissues and organs resulting in MIS-C (21). First, symptoms and signs of MIS-C appear after virus infection, not acutely during COVID-19 (22); second, MIS-C onset lags behind COVID-19 onset by approximately 4–5 weeks, which supports the hypothesis that MIS-C is an immune system disorder associated with SARS-COV-2 infection. A study by Feldstein et al. on 186 patients found that 14 had obvious COVID-19 symptoms before MIS-C onset (21), with an approximate 25-day interval between COVID-19 symptoms and MIS-C onset, and others reporting SARS-CoV-2 infection approximately 1–2 weeks before MIS-C onset. Belot et al. reported that 95 patients with MIS-C were diagnosed post-COVID-19 with a lag time of approximately 4–5 weeks (23). MIS-C, caused by the abnormal immune response in SARS-COV-2 infection, results in multi-organ damage. Gruber et al. reported an immune map of nine cases of MIS-C in which serum antibodies against the S protein on the SARS-CoV-2 surface were detected (24, 25). They found positive serum antibodies in all MIS-C patients, and on evaluating immunoglobulin antibody subtypes, observed increased plasma levels of IgG immunoglobulin antibodies, but decreased levels of plasma IgM antibodies (24). Most patients with MIS-C are positive for anti-SARS-CoV-2 serum antibodies but negative for viral RNA, and only a few patients with MIS-C show PCR positivity. McMurray et al. also found that in most MIS-C cases, PCR nucleic acid detection was negative in most children, with positive serum anti-SARS-CoV-2 antibodies (22). Therefore, these findings show that MIS-C is a type of acquired immune-mediated response following SARS-CoV-2 infection, rather than persistent infection caused by direct virus invasion.

### Direct invasion of the virus

**Figure 2:**
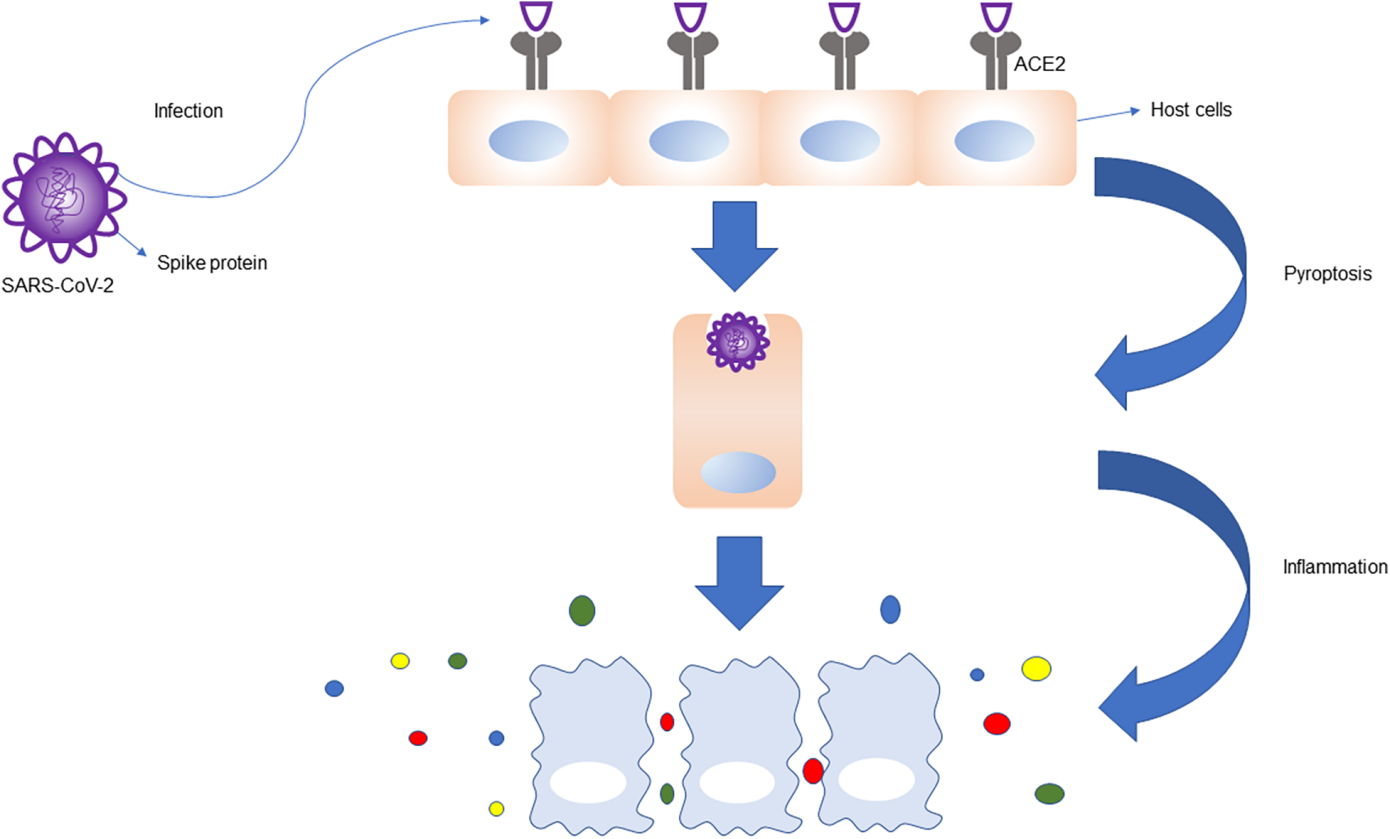
ACE2 receptors are highly expressed in the lung, heart, intestine, vascular endothelial cells, proximal renal tubules, and bladder epithelial cells. S protein on the surface of SARS-CoV-2 binds to ACE2 receptors, which helps the virus to enter the host cells, leading to host cell infection, apoptosis, and inflammation. The immune response is magnified, and the immune cells release a large number of cytokines and chemokines.

Hamming et al. observed that ACE2 receptor expression were higher not only on lung surface and intestinal epithelial cells, but also in cardiomyocytes, indicating that cardiomyocytes are also high-risk targets for novel coronavirus infection (12). The novel coronavirus surface S protein has a high affinity for ACE2 and assists the entry of virus inside host cells by binding with ACE2 (Figure 2). Oudit et al. studied 20 patients who died of SARS-COV infection, and seven of them tested positive for SARS-COV genome in cardiac tissue, suggesting that the virus can directly damage cardiomyocytes (26). SARS-COV-2 and SARS-COV have highly homologous genomes (27, 28). It has been suggested that SARS-COV-2 can also directly invade and injure myocardial cells (29). After binding with ACE2 by S protein, the virus enters cells, where it replicates and destroys host cells in large numbers, and eventually leads to cardiac tissue damage. Rapid replication of the virus may lead to endothelial and epithelial cell apoptosis, blood vessel leakage, and release of proinflammatory regulatory cells and factors.

### Cytokine storm

Many clinical studies have shown that cytokine storms occur in patients with MIS-C (12, 30–36), with upregulation of large numbers of inflammatory cytokines, leading to endothelial cell damage (37, 38). Cytokines are low molecular weight proteins or peptides synthesized and secreted by a variety of cells with many biological activities involved in cell-to-cell signal transduction and interaction. Cytokines have many biological functions, such as innate and adaptive immunity regulation, hematopoiesis, cell growth and differentiation, and tissue repair. Many cytokines promote or restrict each other in the body, forming a complex cytokine immunomodulatory network. Cytokines are double-edged swords; they not only play an immunomodulatory role, but also participate in disease occurrence under certain conditions. They can cause cytokine storm and cytokine storm syndrome, which results in multiple organ damage, function failure, and even death.

Cytokines are produced by many immune cells, including T and B lymphocytes, macrophages, natural killer cells, and dendritic cells. The innate immune system responds to viral invasion by triggering an inflammatory response, activating related signaling pathways, and stimulating interferon (IFN) and other cytokine production. The three most important proinflammatory cytokines in the innate immune response, including interleukin (IL)-1, IL-6, and tumor necrosis factor (TNF)-α, are mainly derived from macrophages, mast cells, epithelial cells, and endothelial cells. The main types and functions of cytokines are as follows: the IL family is responsible for immune cell proliferation and differentiation; IFN regulates innate immunity and activates antiviral properties and anti-proliferation; TNF promotes inflammation and activates cytotoxic T cells; colony-stimulating factor stimulates stem progenitor cell proliferation and differentiation; chemokines mainly control chemotaxis and recruit leukocytes; growth factors promote individual development by promoting cell growth and differentiation.

The main cause of a cytokine storm is immune response imbalance. With disease development, the immune response is dysregulated, and activated T cells or other related immune effector cells release a large number of proinflammatory cytokines and chemokines. These explosive proinflammatory cytokines and chemokines (including IL-1, IL-6, IL-17, IL-12, TNF-α, IFN-α, IFN-β, IFN-γ, and monocyte chemotactic factor 1) lead to the activation of macrophages, mast cells, other immune cells, and endothelial cells; the cytokines released by the latter further activate more immune cells through autocrine and paracrine cascade reactions. This dysregulates the immune regulatory network, forming a cytokines cascade that initiates a cytokine storm, which damages self-immune system homeostasis along with normal tissue and cell function. When cytokine storms occur, inflammatory responses occur in multiple organ systems, which results in pulmonary symptoms (hypoxemia, pulmonary edema caused by vascular leakage, and even acute respiratory distress syndrome), cardiovascular symptoms (hypotension, arrhythmia, myocardial damage, and shock), hematological symptoms (persistent decrease of blood cells, coagulation disorders, and diffuse intravascular coagulation), acute renal injury, multi-organ failure, and even death. This uncontrolled systemic inflammatory response is caused by the release of extreme inflammatory mediators caused by the over-activation and expansion of primary immune cells.

**Figure 3:**
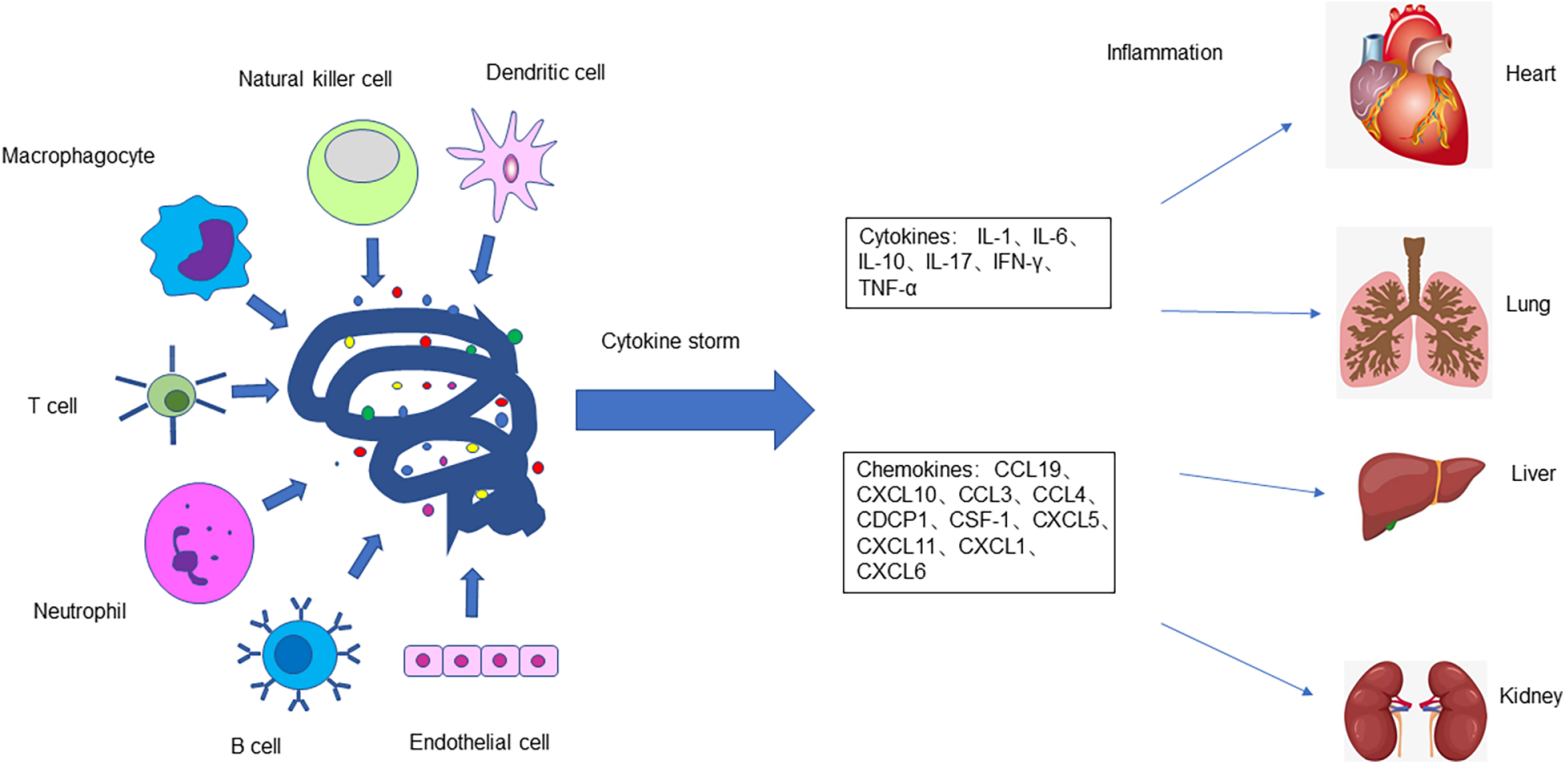
The body's immune response is dysregulated after SARS-CoV-2 infects host cells. T cells, B cells, macrophages, neutrophils, natural killer cells, dendritic cells, epithelial cells, and endothelial cells release a large number of cytokines and chemokines, causing a cytokine storm and severe inflammatory reaction which damages tissues and organs of the body, including the heart, lungs, liver, intestines, kidneys, and testicles.

The expression of ACE2 mentioned above was first found in the heart, kidney, and testis, and later in the upper respiratory tract, lung, intestine, and liver. Cells with ACE2 receptors, such as cardiac cells, can be host cells for SARS-CoV-2 infection. When the virus infects cells, immune cells produce a large number of cytokines. Mast cells regulate the expression of proinflammatory cytokines TNF-α and IL-6, and significantly upregulate the expression of inflammatory factors CCL2, CCL3, CCL5, and CXCL10. Macrophages produce IFN and other cytokines, so a surge of cytokines and chemokines leads to host cell damage, and eventually cardiac damage. Cater et al. (39) analyzed the serum of 25 children with acute stage MIS-C and observed significantly increased levels of IL-1, IL-6, IL-10, IL-17, and interferon-γ. The high expression of human leucocyte antigen (HLA)-DR on T cells in the acute phase indicates that the immune cell group is activated. Consiglio et al. examined plasma samples from 11 children with MIS-C and 28 children with Kawasaki disease and found that elevated IL-6, IL-17A, and CXCL10 play a major role in cytokine storms (40). Gruber et al. (24) reported that the increase in cytokines and chemokines in patients with MIS-C is mainly related to the synthesis and secretion of circulating natural killer cells and T cells, as well as the activation and elevation of CCL19, CXCL10, CCL3, CCL4, CDCP1, and colony-stimulating factor 1. Laura et al. analyzed the immune response in the peripheral blood of patients with SARS-CoV-2 infection and MIS-C, finding obvious activation and proliferation of T cells in patients with MIS-C, with greater activation of CX3CR1 + CD8 + T cells (41). This may explain the cardiac injury caused by cytokine storms in patients with MIS-C (Figure 3).

### Innate and adaptive immune dysregulation

SARS-CoV-2 can activate both innate and adaptive immune responses (42). Viral infection can cause an innate immune response, including elevated erythrocyte sedimentation rate (ESR), C-reactive protein (CRP), serum amyloid A, and ferritin levels, and an increase in some inflammatory cytokines (30, 43, 44). The early response of type-I IFN is the first line of defense in suppressing viral replication and spread; however, SARS-CoV-2 can suppress the response of type-I IFN by interfering with pattern recognition receptors or the type-I IFN receptor-signaling pathway (45). In addition, in genetic immune deficiencies or immunoregulation dysfunction, delayed type-I IFN reaction leads to eosinophil and monocyte macrophage release, followed by apoptosis of T cells, epithelial cells, and endothelial cells. Therefore, the intensity and time point at which the innate immune system responds play a crucial role, and poor regulation of the innate immune response leads to disease progression (46–48).

SARS-CoV-2 infection leads to specific antibody formation that binds host autoantigens. The target antigens of autoantibodies are expressed in the mucosa, heart tissue, and endothelial cells. When antibodies bind to antigens, immune complexes form that activate the viral superantigen series of host immune cells and inflammatory response, promote the activation and release of proinflammatory cytokines (20, 49), and finally lead to heart injury. Gruber et al. found that patients with MIS-C had neutralizing antibodies against SARS-CoV-2, which were related to the activation of IL-18, IL-16, lymphocytes, monocytes, and natural killer cells. Elevated mucosal immune dysregulation (IL-17A, CCL20 and CCL28) and neutrophil and macrophage upregulation of intercellular adhesion molecule 1 and FcγR1 indicate enhanced antigen expression and Fc-mediated response (24). Consiglio et al. analyzed the antigen-antibody responses of patients with MIS-C. They identified autoantibodies, including antibodies to MAP2K2 and the casein kinase family (CSNK1A1, CSNK2A1, and CSNK1E1), which appeared in the acute phase of SARS-CoV-2 infection (40). The targets of antibodies are widely expressed in the heart and other tissues, corresponding to the broad, multisystem involvement in patients with MIS-C.

## Clinical features

The clinical symptoms of cardiac injury in patients with MIS-C mainly include chest pain, arrhythmias, electrocardiogram (ECG) changes, hypotension, and echocardiographic changes (coronary artery dilatation and aneurysms). Laboratory investigations show increased creatine kinase, troponin, and brain natriuretic peptide (BNP) levels. The possible clinical symptoms, laboratory test results, examinations, and imaging changes of cardiac injury are summarized and analyzed in the following sections (Table 2).

**Table 2:** Clinical manifestations, laboratory results, and echocardiography findings in MIS-C patients.

	Zahra (*n* = 35)	Gruber (*n* = 9)	Feldstein (*n* = 186)	Dufort (*n* = 99)	Cheung (*n* = 17)	Shelley (*n* = 8)	Lee (*n* = 28)	Julie (*n* = 21)	Kaushik (*n* = 33)
BNP/pro-BNP increased	35 (100)	8 (88)	94 (50)	74 (74)	17 (100)	5 (62)	12 (42)	14 (66)	16 (48)
Troponin increased	35 (100)	8 (88)	77 (41)	63 (63)	17 (100)	7 (87)	6 (21)	17 (80)	
Arrhythmia	1 (2)	3 (33)			13 (76)	1 (12)		2 (9)	
Ventricular tachycardia					1 (5)				
Ventricular premature beat					1 (5)				
Sinus bradycardia					1 (5)				
Prolonged PR		1 (11)							
Prolonged QT		1 (11)							
ST wave abnormalities	1 (2)	1 (11)			10 (58)				
Hydropericardium	3 (8)			32 (32)	8 (47)			10 (47)	15 (45)
Ventricular dysfunction				51 (51)	17 (100)	7 (87)	11 (39)		
Ejection fraction		3 (33)						16 (76)	21 (63)
<30%	10 (28)								4 (12)
30%–50%	25 (71)								17 (51)
CA dilation/CA aneurysm		6 (17)	15 (8)	9 (9)	1 (5)	1 (12)	6 (21)	5 (23)	
Z > 2	6 (17)							5 (23)	
Z > 2.5			15 (8)	4 (4)					
Reference	(50)	(24)	(21)	(51)	(5)	(1)	(52)	(3)	(53)

A summary of the possible clinical manifestations of MIS-C patients with percentages in parentheses.

BNP, brain natriuretic peptide; CA, coronary artery.

## BNP and troponin

The troponin complex comprises cTnC, cTnI, and cTnT, cTnT and cTnI are unique to myocardial cells and exist in free and bound forms. A small part is soluble and free in the cytoplasm; most of the rest is insoluble and fixed on myofibrils in the form of structural proteins. When the myocardial cell membrane is intact, troponin cannot enter the human blood circulation through the cell membrane, and its quantity in the blood is very low. The free part can enter human blood rapidly only when the integrity of the myocardial cell membrane is damaged. This lays the foundation for the early diagnosis of myocardial injury. BNP is a member of the natriuretic peptide family, which mainly includes atrial natriuretic peptide, BNP, C-type natriuretic peptide, renal natriuretic peptide, and dendroaspis natriuretic peptide. Zahra et al. (50) collected data on 35 patients and found that NT-proBNP or BNP levels increased in all children. Gruber collected data on nine children from New York, and eight were found to have elevated troponin and BNP levels (24). Feldstein et al. reported data on 149 patients with MIS-C with cardiovascular changes in 26 patients; 73% had increased BNP and 50% had increased troponin levels (21). Dufort et al. summarized data on 99 patients who were diagnosed with MIS-C, 74% of whom had elevated troponin levels (51). Huang et al. reported that 12% of patients with COVID-19 in their study were diagnosed with acute myocardial injury, mainly characterized by increased troponin I levels (30). Therefore, elevated BNP or troponin levels in MIS-C patients suggest the existence of myocardial injury.

## Arrhythmia

Under normal conditions, the heart beats regularly within a certain range of frequencies. It originates from the atrionector and conducts to the atrium and ventricle in a certain order and rate. When the heart is damaged, the frequency, rhythm, origin site, conduction velocity, and excitation order of cardiac impulses may be abnormal, resulting in arrhythmia. Whittaker collected data on 58 hospitalized children, of whom four had arrhythmias, including complex tachycardia and conduction block (2). Cheung et al. (5) reported that of 17 children with MIS-C in New York, 16 showed ECG abnormalities; 10 had nonspecific ST/T wave abnormalities, 1 had reduced QRS voltage, and 3 had arrhythmia (ventricular premature contraction, ventricular tachycardia, and sinus bradycardia). Waltuch et al. reported the occurrence of tachycardia in patients with MIS-C (54). Shelley et al. reported arrhythmia in 1 of 8 patients (1). Gruber et al. reported data on nine children from New York, six of whom had significant ECG changes. Therefore, when excluding other arrhythmia causes, arrhythmia after myocardial injury should be noted in patients with MIS-C (24).

## Coronary aneurysm

Coronary aneurysm refers to the local or segmental abnormal expansion of the coronary artery, which is approximately 1.5–2 times larger than the adjacent normal blood vessel or the largest coronary artery. It is a single or multiple phymatoid change, mainly due to the destruction of the elastic layer of the vascular middle membrane, smooth muscle necrosis, vasodilation, and aneurysm formation. In a study on 9 children in New York, echocardiography showed coronary artery dilatation or aneurysm formation in 6 children. Whittaker et al. studied 58 hospitalized children; 55 underwent echocardiography for the assessment of coronary artery aneurysm and 8 had abnormally dilated coronary arteries (*Z*-score >2) including 7 with *Z*-scores greater than 2.5; giant coronary artery aneurysms (*Z*-score >10) were discovered in two patients (2). Toubiana et al. reported that 5 out of 21 children with MIS-C had moderate coronary artery dilatation in France (3). Zahra et al. described six cases (17%) of coronary artery dilatation, including five with left main artery dilatation and one with right coronary artery dilatation (50). Verdoni et al. reported that 2 of 10 children who were diagnosed with MIS-C in Italy during the epidemic had coronary aneurysms on echocardiography (4). Immune system dysregulation and inflammatory cells and cytokines infiltration in patients with MIS-C leads to vascular destruction, smooth muscle necrosis, and coronary artery dilatation or coronary aneurysm.

## Cardiac dysfunction

Cardiac dysfunction refers to impaired ventricular filling and/or ejection fraction caused by various cardiac structural or functional diseases, with cardiac output unable to meet the body's metabolic needs. Based on a literature review, left ventricular systolic dysfunction accounted for a large proportion of cardiac injuries in patients with MIS-C. One study from the UK reported that 75% of children with MIS-C had cardiac dysfunction (1). Rauf et al (55). reported that a 5-year-old Indian boy had left ventricular hypofunction and moderate systolic dysfunction. Verdoni et al. reported that five of ten patients had an ejection fraction of >55% (4). Gema et al. retrospectively analyzed 12 children with SARS-CoV-2 infection and shock; 4 (33%) developed ventricular dysfunction (19). Zahra et al. described left ventricular dysfunction as the main cardiac feature of MIS-C, and persistent diastolic dysfunction as a prominent manifestation of this syndrome. In their study, echocardiography in 35 patients showed decreased left ventricular systolic function on admission, with ejection fraction <30% (10 cases), ejection fraction between 30%–50% (25 cases), left ventricular dysfunction (31 cases), and segmental decreased wall motor function (3 cases); however, right ventricular function was normal in all patients (50). Shubhi et al. studied 33 children with MIS-C; echocardiography showed ventricular ejection fraction <50% in 21 children (65.6%), suggestive of cardiac dysfunction (53).

## Hydropericardium

The normal pericardium is a conical serosal sac composed of visceral and parietal layers. The potential cavity between the parietal layer and the visceral pericardium is the pericardial cavity. A small amount of pericardial fluid has a lubricant role, but if liquid in the pericardial cavity exceeds a certain limit, it is referred to as hydropericardium. In serious cases, this can limit cardiac dilation, reduce cardiac compliance, and hinder diastolic filling of the heart. In MIS-C patients, a few children with heart injury were found to have hydropericardium. Zahra et al. and Verdoni et al. also reported hydropericardium in some children with MIS-C (4, 50).

Guo et al. reported that MIS-C patients with basic cardiac disease are more prone to myocardial injury (56). In patients with cardiovascular diseases such as hypertension, coronary heart disease, and cardiomyopathy, viral diseases can further damage myocardial cells through multiple mechanisms and heart damage is more likely, with increased risk of death. Another study by Shao et al. also reported heart injuries in patients with MIS-C; 30% had a history of coronary heart disease and 60% had hypertension, with raised inflammatory markers such as CRP and leukocytes in patients with myocardial injuries (57). The activation and enhanced release of these inflammatory markers can lead to apoptosis or necrosis of myocardial cells and cause cardiac injury.

In conclusion, the myocardial injury caused by MIS-C is diverse. The most prominent manifestations are increased troponin and BNP levels, ECG changes, and echocardiography findings of coronary artery dilation or aneurysm formation. Some serious cases may include ventricular dysfunction, myocarditis, and heart failure. MIS-C can cause acute thrombotic events or malignant arrhythmias. It can also lead to cardiac arrest and sudden cardiac death in asymptomatic patients or those without congenital cardiovascular disease.

## Treatment

Antiviral and immunoregulatory therapies can be used for myocardial injury treatment. One can also use cytokines involved in the pathogenesis of biological therapies. Immunoregulatory therapies include intravenous immunoglobulin (IVIG) and corticosteroids. Biological agents include IL-1, IL-6 and TNF-α receptor antagonists. Based on the clinical symptoms and signs of myocardial injury, symptomatic supportive treatment should be administered. Belhadjer et al. (50) reported that early diagnosis and treatment with IVIG and corticosteroids improved the clinical path of multisystem diseases, which may prevent ventricular dysfunction and reduce heart failure severity. Therefore, almost all patients with MIS-C in their study used gamma globulin and corticosteroids in the early stages, which prevented serious cardiac injury, including coronary aneurysm formation and heart failure. For patients with a persistent inflammatory response, biological agents can be used to reduce cardiac injury. Patients with cardiac dysfunction can be treated with vasoactive agents, assisted mechanical ventilation, and veno-arterial extracorporeal membrane oxygenation (VA-ECMO). Few studies have shown good prognosis in patients with MIS-C who were treated with IVIG and corticosteroids together (Table 3). In addition, most children treated with biological agents (including IL-1, IL-6, and TNF-α inhibitors) improved clinically, and were discharged. Furthermore, for patients with shock and decreased circulatory function, symptomatic supportive treatments such as vasoactive agents, mechanical ventilation, and VA-ECMO can be used.

**Table 3:** MIS-C patient-related treatments.

	Zahra (*n* = 35)	Gruber (*n* = 9)	Feldstein (*n* = 186)	Dufort (*n* = 99)	Cheung (*n* = 17)	Shelley (*n* = 8)	Pui (*n* = 28)	Julie (*n* = 21)	Shubhi (*n* = 33)
IVIG	25 (71)	8 (88)	144 (77)	69 (69)	13 (76)	8 (100)	20 (71)	21 (100)	18 (54)
Corticosteroids	12 (34)		91 (48)	63 (63)	15 (88)	4 (50)	17 (60)	10 (47)	17 (51)
IL-1 receptor antagonist	3 (8)		24 (12)				5 (17)		4 (12)
veno-arterial extracorporeal membrane oxygenation (VA-ECMO)	10 (28)								
IL-6 receptor antagonist		9 (100)	14 (7)		1 (5)				
TNF-α receptor antagonist						1 (12)			12 (36)
Reference	(50)	(24)	(21)	(51)	(5)	(1)	(52)	(3)	(53)

A summary of the MIS-C patient-related treatments with the percentage of patients in parentheses.

IVIG, intravenous immunoglobulin; IL-1, interleukin 1; VA-ECMO, veno-arterial extracorporeal membrane oxygenation; IL-6, interleukin 6; TNF, tumor necrosis factor.

## IVIG

IVIG has a strong effect on immune substitution and regulation (58), has anti-inflammatory properties, and can neutralize bacterial toxins (59). IVIG regulates innate and adaptive immunity through various mechanisms. The symptoms of patients with MIS-C who used IVIG can be relieved quickly, due to a significant decrease in cytokines, monocytes, macrophages, neutrophils, and activated T cells, and increased natural killer cells.

## Corticosteroids

Corticosteroids affect the survival and activity of immune cells and the expression of inflammatory markers through many molecular mechanisms. Corticosteroids inhibit transcription of proinflammatory cells in the signaling pathway, resulting in reduced release of proinflammatory cytokines (IL-1, TNF-α, IL-6, IL-11, IL-16). It restrains some of the stimulators involved in neutrophils, antigen presenting cells, and lymphocyte activation and differentiation (60, 61). In addition, corticosteroids affect somatic and endothelial cells by downregulating the accumulation of adhesion molecules and neutrophils, and inhibiting platelet adhesion and T and B lymphocytes activity (16, 62, 63). Corticosteroid use is not recommended in the early stages of MIS-C as they might delay virus clearance and increase infection risk (59).

## Biological agents

The biological response modifier drug is a new therapeutic agent composed of recombinant human monoclonal antibody or receptor antagonists, including IL-1 receptor antagonists, IL-6 receptor antagonists, and TNF-α receptor antagonists. It has been reported that the application of biological agents can inhibit cytokine storms in patients with MIS-C and improve clinical symptoms, signs, and inflammatory responses.

## IL-1 receptor antagonist

Anti-IL-1 antagonist is the first-line biological agent for MIS-C treatment. IL-1 is a pleiotropic cytokine that is activated through the engagement of endothelial cells and other cells involved in the innate immune response. After activation, expression of IL-6, IL-8, TNF, IL-10, and vascular endothelial cell adhesion molecules increases through the hydroxybenzoyl mechanism (16). IL-1 is the main immunomodulator of inflammatory cytokine storms. IL-1 receptor antagonists can downregulate the inflammatory response secondary to IL-1, decrease adhesion molecules on endothelial cells, and inhibit vascular wall damage caused by neutrophil activation and degranulation (16). In a clinical study of Kawasaki disease, anakinra, an IL-1 receptor antagonist, was found to reduce coronary aneurysm development and myocarditis (64). In sample data from nine patients, anakinra was found to improve clinical manifestations and laboratory investigations effectively (65). In another study, it was found that the application of anakinra could effectively reduce patients' demand for assisted mechanical ventilation and decrease mortality in the intensive care unit (66).

## IL-6 receptor antagonist

IL-6 is the main proinflammatory cytokine, leading to increased levels of cytokines such as IL-2, IL-8, IL-10, and TNF-α. Data suggest that the IL-6 pathway may play an important role in the inflammatory response of patients with COVID-19 (59). Therefore, it may be a potential target for immunotherapy in patients with MIS-C. Tocilizumab is an IL-6 receptor antagonist that downregulates IL-6's paracrine and autocrine functions and prevents inflammatory response events, including CD4 + T cells differentiation and T regulatory cell inhibition (67). Tocilizumab is approved for systemic juvenile idiopathic arthritis in children aged 2 years and older. This disease has a similar cytokine cascade response to MIS-C; therefore, the study of tocilizumab in the treatment of MIS-C patients is promising (68, 69). One study showed that during tocilizumab administration, 75% of patients with severe COVID-19 had fever cessation, lower oxygen requirements, and significantly improved computed tomography imaging changes (70). Early clinical reports showed that after the application of tocilizumab, 20 severely ill patients had fever subside within 1 min, and 95% of them recovered well enough to be discharged within 2 weeks. Guo et al. showed that tocilizumab treatment can improve the excessively activated inflammatory immune response in children and enhance the antiviral immune response regulated by B cells and CD8 + T cells (56). Luo et al. (71) suggested repeated treatment with a low-dose IL-6 receptor blocker to obtain the greatest effect. Zain et al. (72) reported that patients who received tocilizumab treatment had marked improvements in oxyhemoglobin saturation, lung imaging, ventilator support time, CRP, ferritin, and LDH, among other factors. In summary, IL-6 receptor antagonists are a promising target for MIS-C treatment, and further research should be conducted on their efficacy and safety.

## TNF-α receptor antagonist

TNF-α is considered to be a key participant in the production of proinflammatory cytokines and the activation of platelets and is involved in the occurrence of many chronic autoinflammatory diseases. Since TNF-α plays an important role in the activation of the cytokine pathway, it is also a key biological target (73). Infliximab is a TNF-α antagonist that can be used to treat patients with MIS-C. As TNF-α antagonists are an effective treatment for Crohn disease, some studies have shown some success in treating patients with MIS-C and Crohn disease at the same time (74). There is still a lack of experience with the combined use of anti-IL-1 and anti-TNF-α. Future studies should focus on determining whether drug combinations maximize treatment efficacy while minimizing side effects.

## Targeted blocking drugs for ACE2 receptor

When the virus invades, the S protein on the virus surface binds to ACE2 on the surface of the target cell. In view of this mechanism, it is possible to study targeted blocking drugs for the S protein ACE2 receptor. ACE2 derivatives (P4, P5, and P6) are now in commercial use as they are effective in blocking SARS-CoV invasion. However, the efficacy of these drugs is still unclear and many drugs are in different phases of clinical trials. In addition, we should also consider the physiological changes to RAS induced by these drugs, which may lead to secondary tissue injury. All these factors require further study.

## Conclusion

The clinical manifestations of myocardial injury caused by MIS-C are diverse. This review article summarizes the clinical manifestations, laboratory investigations, and treatments for cardiac injury. This may help clinicians in early diagnosis and treatment to reduce complications, severity of heart injury, and mortality. Studies on the treatment of this disease is very limited. For this reason, the therapeutic effect of the treatments on cardiac injury cannot be assessed. In addition, the long-term consequences of cardiac damage due to MIS-C disease are unknown. Some of the treatments described in this article need further study.
